# (−)-Epigallocatechin-3-Gallate Ameliorates Learning and Memory Deficits by Adjusting the Balance of TrkA/p75^NTR^ Signaling in *APP/PS1* Transgenic Mice

**DOI:** 10.1007/s12035-013-8608-2

**Published:** 2013-12-20

**Authors:** Mingyan Liu, Fujun Chen, Lei Sha, Shuang Wang, Lin Tao, Lutian Yao, Miao He, Zhimin Yao, Hang Liu, Zheng Zhu, Zhenjie Zhang, Zhihong Zheng, Xianzheng Sha, Minjie Wei

**Affiliations:** 1 Department of Pharmacology, School of Pharmaceutical Sciences, China Medical University, No.92 Bei’er Road, Heping District, Shenyang, 110001 Liaoning Province People’s Republic of China; 2 Laboratory Animal Center, China Medical University, Shenyang, People’s Republic of China; 3 Department of Biomedical Engineering, College of Basic Medical Science, China Medical University, Shenyang, People’s Republic of China

**Keywords:** EGCG, NGF, TrkA/p75^NTR^ balance, *APP/PS1* transgenic mouse, Learning and memory deficits

## Abstract

Alzheimer's disease (AD) is pathologically characterized by deposition of β-amyloid (Aβ) peptides, which closely correlates with the balance of nerve growth factor (NGF)-related TrkA/p75^NTR^ signaling. (−)-Epigallocatechin-3-gallate (EGCG) is used for prevention and treatment of many neurodegenerative diseases, including AD. However, whether the neuroprotective effects of EGCG treatment were via modulating the balance of TrkA/p75^NTR^ signaling was still unknown. In this study, we found that EGCG treatment (2 mg · kg ^–1^ · day ^–1^) dramatically ameliorated the cognitive impairments, reduced the overexpressions of Aβ(1–40) and amyloid precursor protein (APP), and inhibited the neuronal apoptosis in the *APP/PS1* mice. Interestingly, the EGCG treatment enhanced the relative expression level of NGF by increasing the NGF/proNGF ratio in the *APP/PS1* mice. Moreover, after EGCG treatment, TrkA signaling was activated by increasing the phosphorylation of TrkA following the increased phosphorylation of c-Raf, ERK1/2, and cAMP response element-binding protein (CREB), simultaneously the p75^NTR^ signaling was significantly inhibited by decreasing the p75^ICD^ expression, JNK2 phosphorylation, and cleaved-caspase 3 expression, so that the Aβ deposits and neuronal apoptosis in the hippocampus were inhibited.

## Introduction

Alzheimer's disease (AD), an age-related neurodegenerative disorder, is the predominant form of dementia in the elderly. AD is clinically characterized by cognitive impairment and pathologically characterized by extracellular senile plaques largely composed of β-amyloid (Aβ) peptide and neuronal loss [1–3]. The abnormal accumulation of Aβ generated by successive proteolysis of β-amyloid precursor protein (APP) causes the neurotoxic effects by oxidative stress, calcium overburden, and neuronal apoptosis in vitro and in vivo, and is one of the main neuropathological hallmarks of AD [4–8]. The decreased activity of cell survival and neuronal apoptosis is the final destiny of neurons in AD and other neurodegenerative diseases. Nerve growth factor (NGF), the first neurotrophin known for its stimulatory effects on differentiation, maintenance, survival, and plasicity of brain function, elicits most of the survival and growth properties mediated by activating the tyopomyosin kinase receptor A (Trk A) related signaling pathway and inhibiting the p75 neurotrophin receptor (p75^NTR^) related signaling pathway [9–13]. It was reported that the NGF deficiency would lead to a TrkA/p75^NTR^ imbalance in AD brain, which might be one of possible mechanisms of neurodegeneration. When NGF was deficient in AD brain, the TrkA-related signaling was inhibited and the p75^NTR^-related signaling was activated concurrently. Then, the expression levels of memory-, learning- and apoptosis-related proteins downstream were affected, and then, the neuronal degeneration, cell death/loss, and cognitive deficits occurred [10, 11, 14–16]. Therefore, it is meaningful to find a way to ameliorate the low expression of NGF and to adjust the TrkA/p75^NTR^ imbalance in AD brain.

(−)-Epigallocatechin-3-gallate (EGCG) is the most abundant polyphenolic constituent in green tea and was particularly suggested to have potent ironchelating, antioxidant, anti-inflammatory, and anticancer effects [17–22]. Previous studies reported that EGCG treatment significantly reduced Aβ generation in human-derived neuroblastoma cell line (SH-SY5Y) induced by 3-HK, in murine neuron-like cells (N2a) transfected with human “Swedish” mutant APP, and in primary neurons derived from Swedish mutant APP-overexpressing mice (Tg*APP*_*sw*_ line 2576), and markedly improved the cognitive deficits, APP processing, and Tau pathology in D-gal-induced AD mice, Tg*APP*_*sw*_ line 2576 transgenic mice and *PS2* transgenic mice, suggesting that EGCG treatment exerted the neuroprotective effects in vitro and in vivo [23–27]. However, there is no study performed to evaluate whether EGCG treatment ameliorates cognitive deficits in *APP/PS1* transgenic mice and, if it does, whether the ameliorating effect is through regulating the balance of TrkA/p75^NTR^ signaling. Therefore, in the present study, we examined the neuroprotective effects of EGCG treatment in APP/PS1 transgenic mice on the neurodegenerative pathology and behavioral deficits firstly. Furthermore, the possible neuroprotective mechanisms on the balance of NGF-related TrkA/p75^NTR^ signaling after EGCG treatment in *APP/PS1* transgenic mice were investigated.

## Methods

### Animals and Treatment

*APP/PS1* double-transgenic mice were obtained from The Jackson Laboratory [*B6.Cg-Tg (APPswe, PSEN1dE9) 85Dbo/J*]. These mice express a chimeric mouse/human *APP* containing the *K595N/M596L* Swedish mutations and a mutant human *PS1* carrying the exon 9-deleted variant under the control of mouse prion promoter elements, directing transgene expression predominantly to central nervous system (CNS) neurons [28–30]. The offspring 9-month-old double-transgenic mice overexpressing *APP/PS1* were compared with their wild-type (WT) littermates so that age and background strain were matched. Males and females were equally distributed into each group.

All mice were housed in cages in a controlled environment (22–25 °C, 55 % relative humidity, 12 h light/dark cycle) with free access to food and water, and maintained in a specific pathogen-free environment at Laboratory Animal Science of China Medical University. All animal care and experimental procedures were in compliance with the Standard Medical Laboratory Animals' Care and Use Protocols (Ministry of Health PR China, 1998) and the Laboratory Animal Ethical Standards of China Medical University.

EGCG (Sigma-Aldrich Chemicals Pvt. Ltd., USA) was dissolved into distilled water at a concentration of 0.2 mg/mL. Distilled water was used as vehicle. EGCG or vehicle was administered by intragastric administration (2 mg/kg body weight) at 9:00 am once daily for 4 weeks. Twenty 9-month-old *APP/PS1* transgenic mice were randomly and equally assigned into two groups, EGCG-treated *APP/PS1* group and vehicle-treated *APP/PS1* group (*APP/PS1* group), with 10 mice in each group. Another 10 wild-type littermates were assigned as aging-control group (WT group). The EGCG-treated *APP/PS1* group intragastically received EGCG (2 mg · kg ^–1^ · day ^–1^) for 4 weeks; the *APP/PS1* and WT groups received the same volume of vehicle for 4 weeks.

### Passive Avoidance Test

After 4 weeks of the EGCG treatment, mice were subjected to a passive avoidance test (PAT), as previously described [31, 32]. The apparatus (BA-200, Chengdu Taimeng Tech. Co. Ltd., Chengdu, People's Republic of China) consists of two compartments with a lighted compartment connected to a darkened one by an automatic guillotine door. The dark compartment is equipped with a grid floor placed through which a foot-shock can be delivered. In the training session, each mouse was placed in the light compartment and allowed to explore for 3 min, at which point the guillotine door was raised to allow the mouse to enter the dark compartment. When the mouse entered the dark compartment, the guillotine door was closed and an electrical foot shock (0.5 mA, 1 s duration) was delivered. Training session was conducted before the test session. The test session was performed 24 h after the training session. In the test session, each mouse was placed in the light compartment and allowed to explore 3 min, and then the guillotine door was raised. The latencies and frequencies for mice to enter the dark compartment were recorded during the whole testing period (300 s).

### Morris Water Maze Test

After the PAT, mice were given another behavioral test, Morris water maze (MWM), for consecutive 7 days including navigation tests and a probe trial test, as previously described with a few modifications [33, 34].

The Morris water maze is a stainless-steel circular water tank (120 cm diameter × 50 cm height) equipped with a platform (10 cm diameter) placed in the second quadrant and submerged 0.5–1 cm below the surface of water. In brief, mice were allowed to swim freely for 1 min without the platform to adjust themselves to the circumstances at the baseline day (day 0). From the first day to the fifth day, the platform was placed under the water in the tank for navigation tests, and each mouse was subjected to four trials per day at an intertrial interval of 60 s for spatial acquisition. Different start locations were used on each trial. If a mouse failed to find the platform within 60 s, it would be picked up and placed on the platform for 60 s. For each trial, the latency and the path length by which the mouse found the hidden platform were recorded. On the sixth day, a probe trial was performed to assess memory consolidation. In this trial, the platform was removed from the tank, and the mice were allowed to swim freely for 60 s. The start position was a novel one which was 180° from the original platform position to ensure that the spatial preference was a reflection of the memory of the goal location rather than for a specific swim path. The frequency that each mouse crossed the center of the quadrant (where the platform was previously located) and the percent of time that each mouse spent in the quadrant were recorded. All the data were obtained by a video tracking system (Chengdu Taimeng Tech. Co. LTD, Chengdu, People's Republic of China).

### Locomotivity Test

Locomotivity test were conducted after MWM and PAT using a locomotivity testing paradigm (ZZ-6 system for mice, Chengdu Taimeng Tech. Co. Ltd., Chengdu, People's Republic of China). Briefly, mice were placed in the system and the exploration was assessed for 10 min. Cages were routinely cleaned with ethanol following each session. The locomotivity and the frequency of stand-up for each mouse were recorded.

### Animal Dissection and Tissue Collection

Twenty-four hours after the behavioral tests, half of the mice (*n* = 5) in each group were anesthetized with sodium pentobarbital (50 mg/kg) by intraperitoneal administration and then transcardially perfused with normal saline followed by 4 % paraformaldehyde solution. The brains were removed and postfixed in 4 % paraformaldehyde overnight at 4 °C, and routine paraffin sections (4 μm) were prepared for TUNEL staining and immunohistochemistry analysis. The rest half of the mice in each group were killed by decapitation, and the brains were rapidly removed and cut sagittally into left and right hemispheres on an ice-cooled board 24 h after the behavioral tests. The hippocampus and cerebral cortex were dissected out and stored at −80 °C for Western blotting analysis.

### Immunohistochemistry

Sections were dewaxed in xylene and rehydrated in a series of graded alcohols. After dewaxing and rehydration, sections were boiled in citrate buffer (10 mM, pH 6.0) for 20 min by microwave oven for antigen retrieval. Then, the sections were treated with 3 % H_2_O_2_ in 0.1 M phosphate buffered solution (PBS) for 20 min at room temperature to abolish endogenous peroxidase activity. After washing with PBS, sections were blocked with normal goat serum at 37 °C for 30 min. Subsequently, sections were incubated overnight at 4 °C with primary rabbit anti-caspase 3 antibody (1:500, Abcam), anti-Aβ antibody and anti-APP antibody (1:500, Thermo). After rinsing, sections were incubated in biotinylated goat anti-rabbit IgG (1:200, Maixin) at 37 °C for 30 min, followed by streptavidin–peroxidase conjugate (1:200, Maixin) at 37 °C for 30 min. The immunoreactions were visualized by staining sections with 3′-diaminobenzidine (DAB) for 1–3 min. Finally, the sections were counterstained with hematoxylin for 5 min.

### TUNEL Staining

TUNEL staining was performed on the paraffin-embedded sections using the in situ cell death detection kit (Promega, Madison, USA). According to the standard protocols provided by manufacturer, the sections were deparaffined, rehydrated in graded alcohol series, and treated with 3 % H_2_O_2_ for 10 min at room temperature. Then, these sections were rinsed three times with PBS, incubated in a 20 μg/ml Proteinase K working solution for 15 min at 37 °C, and rinsed with PBS thrice followed by incubation with TUNEL reaction mixture for 2 h at 37 °C. Biotinylated antidigoxigenin antibody was then reacted with the sections for 30 min at 37 °C. Apoptotic nuclei were visualized using the peroxidase-DAB reaction. The sections were counterstained with hematoxylin. TUNEL-positive neurons in hippocampus were counted in six random areas of sections per high-power field (×400), and represented as the percentage of 100 neurons as the cell apoptosis index.

### Fluoro-Jade B Staining

Tissue sections were mounted on 2 % gelatin-coated slides and then air-dried overnight. The slides were immersed in 100 % ethanol for 5 min, 80 % ethanol for 5 min, 70 % ethanol for 2 min, and distilled water for 2 min. After incubation in 0.06 % solution of potassium permanganate for 15 min, the sections were rinsed in distilled water for 2 min, and then incubated in 0.0004 % Fluoro-Jade B (Chemicon) staining solution in 0.1 % acetic acid for 30 min, rinsed again in distilled water for 1 min for three times, and air-dried. Finally, the dry slides were cleared by immersion in xylene for 1 min before coverslipping with DPX (Fluka). Fluoro-Jade B positive cells per square millimeter in hippocampus were counted in six random areas of sections per high-power field (×400).

### Image Analysis

Images were obtained using an Olympus BX-61 microscope and digitized using an attached Spot flex imaging system (Diagnostic). Images of 4-μm sections through each anatomic region of interest (hippocampus or cortical areas) were captured. The related studied indexes were assessed by densitometric measurements of product on digitized images. Regions of interest were identified under dark-field illumination. Background level was determined from the area outside the brain section. First-level threshold was determined after subtracting the background level. The second level threshold was determined after subtracting nonspecific binding in omit and negative controls. Thus, the net integrated optical density representing specific immunoreactive signal was determined after subtracting first and second level thresholds. Each sample was digitized under identical illumination, threshold, and camera settings. The data were analyzed with an image analysis system (Image-Pro Plus 6.0).

### Western Blot

Hippocampus samples were minced into small pieces, homogenized on ice in precooling RIPA buffer containing 150 mM NaCl, 50 mM Tris–HCl (pH 8.0), 1 % NP-40, 0.5 % sodium deoxycholate, 0.1 % sodium dodecyl sulfate (SDS), 0.1 % phenylme-thylsulfonyl fluoride, and incubated overnight at 4 °C. The homogenate was centrifuged at 12,000×*g* for 30 min at 4 °C, and the supernatant was divided into aliquots and frozen at −80 °C. Protein qualification was carried out using a BCA kit (Walterson Biotechnology Inc., Beijing, China). The total protein extract (40 μg per lane) was separated on SDS-polyacrylamide gels, and transferred onto polyvinylidene difluoride membranes (Millipore, CA). Nonspecific binding sites on the membrane were blocked by PBS containing 0.1 % Tween-20 (PBST) with 5 % bovine serum albumin for 1 h. The membranes were then incubated overnight at 4 °C with specific primary antibodies. Rabbit polyclonal antibodies against NGF (1:500, Santa Cruz), APP and Aβ1-40 (1:1,000, Sigma), TrkA and phospho-TrkA (Tyr490) (1:1,000, Cell Signaling), c-Raf and phospho-c-Raf (Ser 338) (1:2,000, Cell Signaling), ERK1/2 and phospho-ERK1/2(Thr202/Tyr204) (1:2,000, Cell Signaling), cAMP response element-binding protein (CREB) and phospho-CREB (Ser 133) (1:1,000, Cell Signaling), p75^ICD^ (1:500, Santa Cruz), JNK2 and phospho-JNK2 (Tyr 185) (1:1,000, Cell Signaling), cleaved-caspase 3 (Asp 175) (1:1,000, Cell Signaling), p53 (1:500, Santa Cruz), and β-actin (1:1,000, Santa Cruz), were used in this study. After washed with PBST, the membranes were incubated with goat horseradish peroxide-conjugated second rabbit anti-body (1:2,000; Santa Cruz) for 1 h at room temperature. Immunoreactive bands were visualized using the enhanced chemiluminescent kit (ECL+, Amersham Biosciences). The bands were measured using the Quality One analysis software (BioRad), and the intensity of phophos-immunoreactive protein bands was normalized to total protein. The immunoreactive protein bands of total protein were normalized to β-actin.

### Statistical Analysis

Data were expressed as means ± standard deviation (SD). All statistical analysis was performed using SPSS (Statistical Package for the Social Sciences, version 13.0) statistical software package. Differences among WT, *APP/PS1*, EGCG-treated *APP/PS1* groups were assessed by one-way ANOVA followed by Turkey's test. *P* < 0.05 was considered significant.

## Results

### EGCG Treatment Ameliorated Learning and Memory Impairment in *APP/PS1* Mice

To investigate whether EGCG treatment improved learning and memory in *APP/PS1* mice, behavioral tests such as PAT, MWM, and locomotivity test were performed following EGCG treatment.

**Fig. 1:**
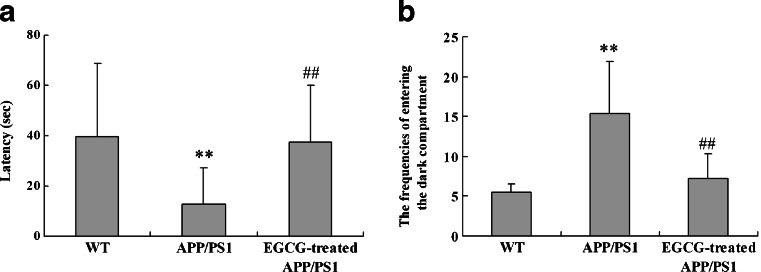
EGCG treatment ameliorated the latency (**a**) and the frequencies of entering the dark compartment (**b**) of *APP/PS1* mice in the passive avoidance test (*n* = 10) **P < 0.01, compared with WT group; ^##^ *P* < 0.01, compared with *APP/PS1* group

In PAT, the learning and memory performance was evaluated by the latency and the frequency of entering the dark compartment in WT, *APP/PS1*, and EGCG-treated *APP/PS1*groups. Compared with WT group, the latency to enter the dark compartment in the *APP/PS1* group was significantly decreased (*P* < 0.01, Fig. 1a), and the frequency of entering the dark compartment was increased significantly (*P* < 0.01, Fig. 1b), suggesting that the learning and memory performance of *APP/PS1* transgenic mice were impaired. However, in the EGCG-treated *APP/PS1* group, the latency to enter the dark compartment was apparently increased (*P* < 0.01, Fig. 1a) and the frequency of entering the dark compartment was decreased significantly (*P* < 0.01, Fig. 1b) compared with the *APP/PS1* group, suggesting that EGCG treatment ameliorated the learning and memory impairment in *APP/PS1* mice.

**Fig. 2:**
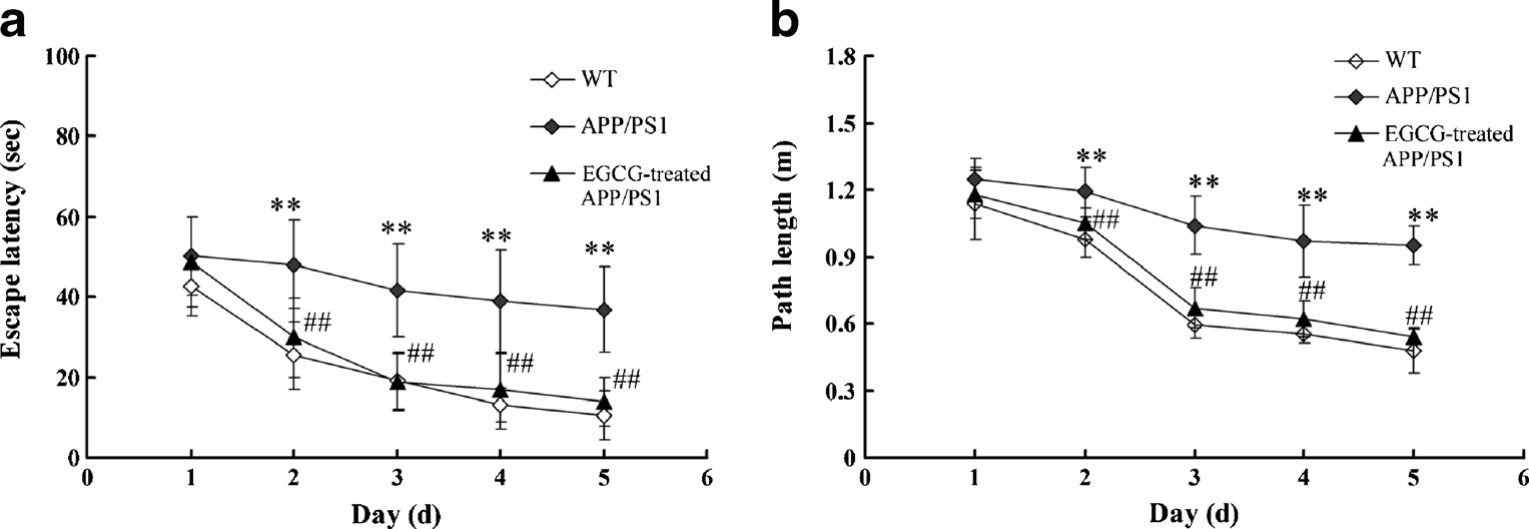
EGCG treatment improved the escape latency (**a**) and path length (**b**) of *APP/PS1* mice in the navigation test of Morris water Maze (*n* = 10). *P < 0.05, **P < 0.01, compared with WT group; ^#^ *P* < 0.05, ^##^ *P* < 0.01, compared with *APP/PS1* group

**Fig. 3:**
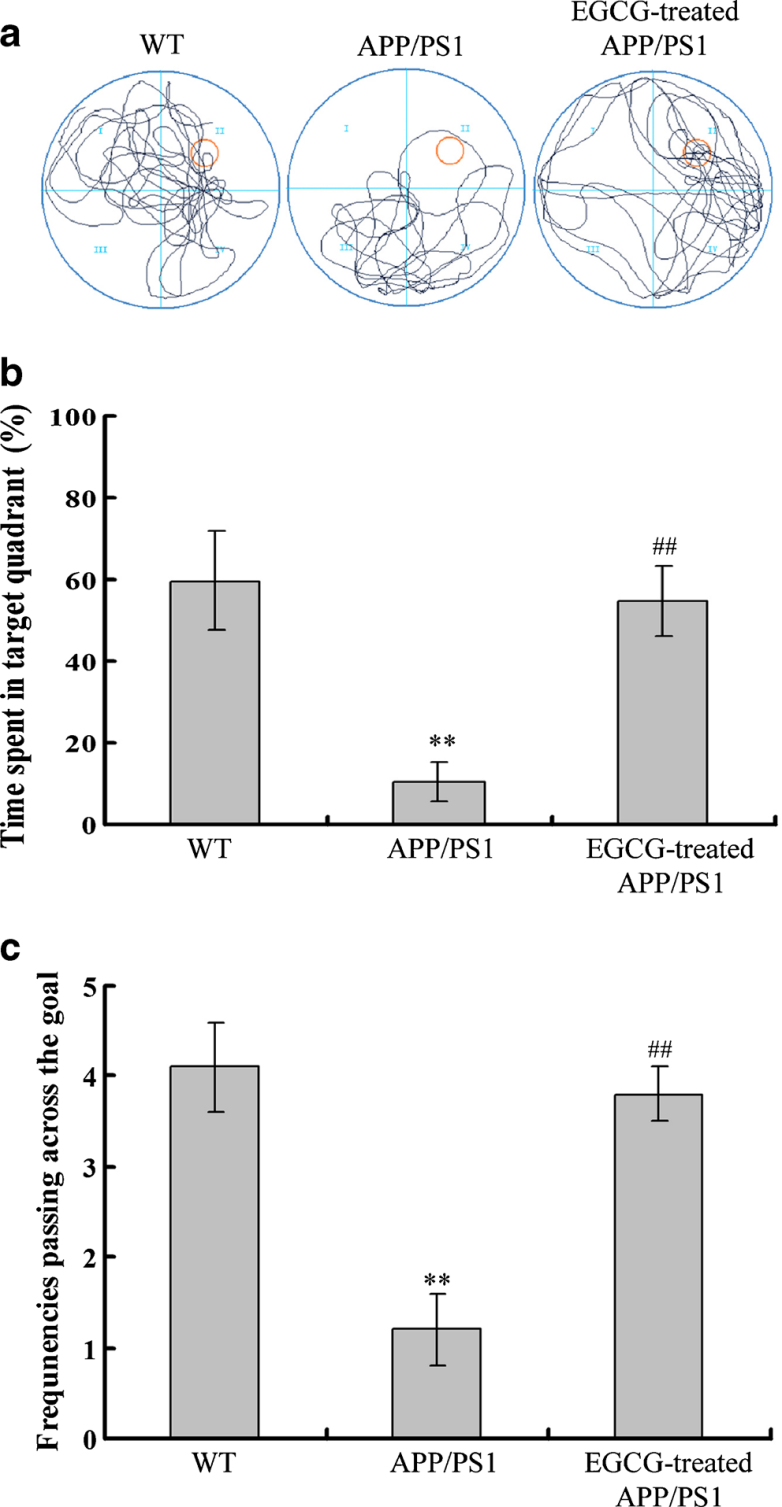
EGCG treatment improved the performance of the probe trial in Morris water maze of *APP/PS1* mice (*n* = 10). **a** The representive locus plot after EGCG treatment in the probe trial. **b** EGCG treatment prolonged the time spent in target quadrant. **c** EGCG treatment increased the frequencies of passing through the goal. *P < 0.05, **P < 0.01, compared with WT group; ^#^ *P* < 0.05, ^##^ *P* < 0.01, compared with *APP/PS1* group

Then, the MWM test was conducted. In the navigation tests of MWM test, on the first day, we observed that the WT mice, *APP/PS1* mice and EGCG-treated *APP/PS1* mice had a similar escape latency (*P* > 0.05, Fig. 2a) and path length (*P* > 0.05, Fig. 2b), indicating that the motility or vision of mice was not affected before the navigation test and probe trial. In the navigation tests of the following days (from the second day to the fifth day), the *APP/PS1* group showed higher escape latency (*P* < 0.01, Fig. 2a), longer path length (*P* < 0.01, Fig. 2b) and slower improvement than the WT group. While the escape latency and the path length in the EGCG-treated *APP/PS1*group in the navigation training were significantly improved, compared with those in the *APP/PS1* group (*P* < 0.01, Fig. 2a and b), suggesting that EGCG treatment improved the impairments of spatial acquisition of *APP/PS1* mice. Moreover, in the probe trial on the sixth day of MWM tests, the *APP/PS1* mice passed through the original position of the platform with fewer times (*P* < 0.01, Fig. 3a and c) and had shorter stay in the target quadrant (*P* < 0.01, Fig. 3a and b) than the WT group. In the EGCG-treated *APP/PS1* group, the time spent in target quadrant (*P* < 0.01, Fig. 3a and b) and the frequencies to pass the goal (*P* < 0.01, Fig. 3a and c) significantly increased compared with the *APP/PS1* group. The MWM test results indicated that EGCG treatment improved the impairments of memory consolidation of *APP/PS1* mice.

**Fig. 4:**
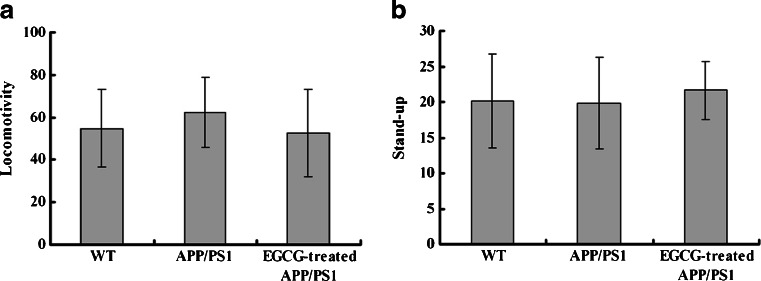
EGCG treatment did not affect locomotivity (**a**) and the frequencies of stand-up (**b**) of APP/PS1 mice in locomotivity test (*n* = 10). *P* > 0.05 among all the groups

In order to investigate whether the behavioral deficits were affected by the locomotor activity among different groups, we studied the locomotivity and the frequencies of stand-up in all groups. We found that there were no significant differences among the groups (*P* > 0.05, Fig. 4a and b), suggesting that it was the memory and learning ability, not locomotivity, contributing to the changes of behavioral deficits in PAT and MWM.

Thus, these results of behavioral tests demonstrated that EGCG treatment ameliorated the cognitive deficits in spatial learning and memory function of *APP/PS1* mice.

### EGCG Treatment Decelerated Aβ(1–40) Plaque Formation and Reduced APP Expression in the Brains of *APP/PS1* Mice

Behavioral test showed that the mice in *APP/PS1* group had impairments of cognition, and the impairments were rescued with EGCG treatment. Next, we investigated the Aβ(1–40) plaque formation and APP expression in the *APP/PS1* groups to ensure these mice suffering a similar pathological process of AD and tested the Aβ(1–40) plaque formation and APP expression in the EGCG-treated *APP/PS1* mice to verify the effect of EGCG treatment.

**Fig. 5:**
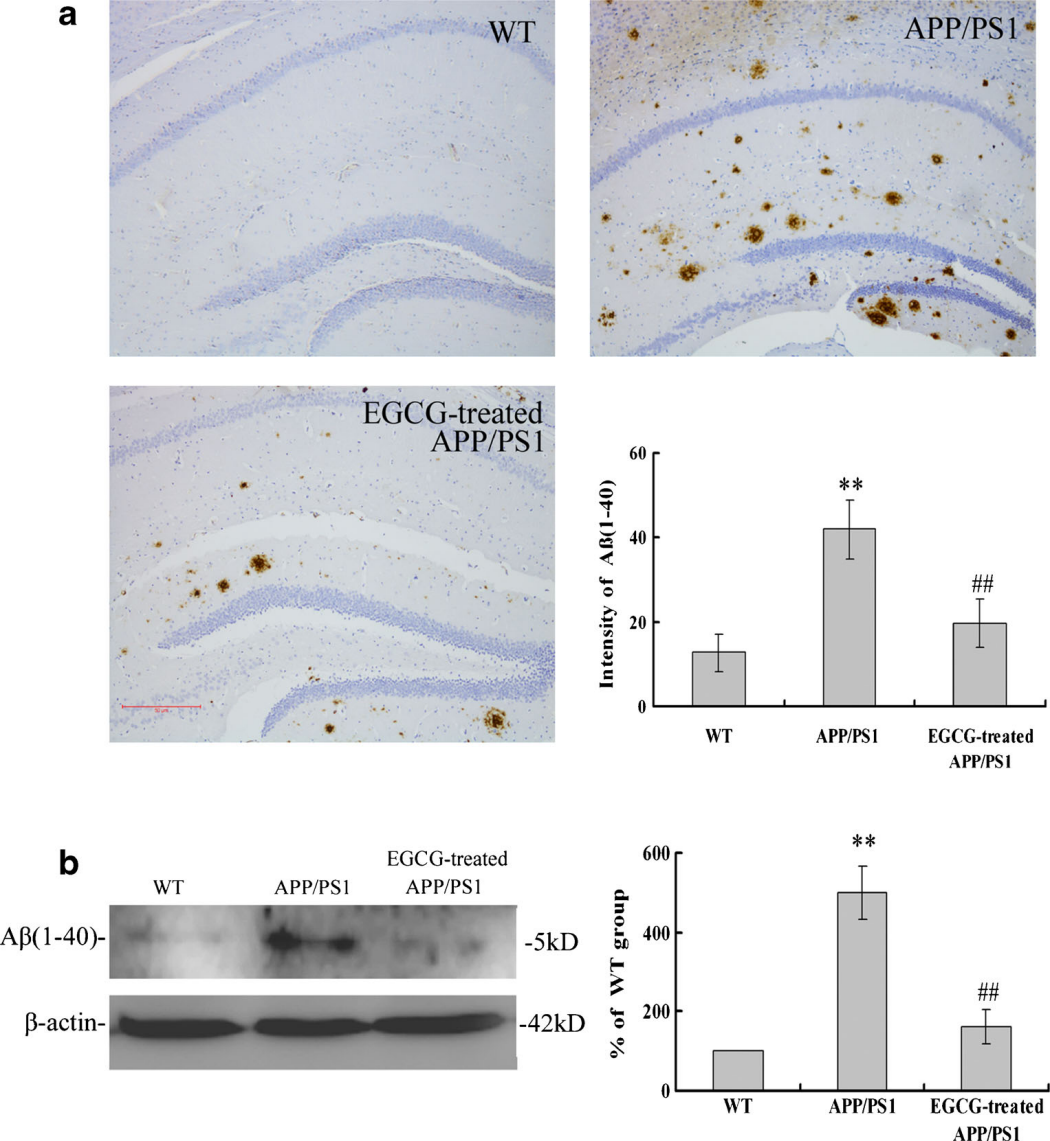
EGCG treatment ameliorated the overexpression of Aβ(1–40) in the hippocampus of *APP/PS1* mice by immunohistochemical staining (**a**) and Western blot (**b**) (*n* = 5). **P < 0.01, compared with WT group; ^##^ *P* < 0.01, compared with *APP/PS1* group

The formation level of Aβ(1–40) plaque in the hippocampus of *APP/PS1* mice was measured with imunohistochemistry methods (Fig. 5a) and Western blot (Fig. 5b). After a 4-week administration of EGCG, the desposition of Aβ(1–40) plaques in the EGCG-treated *APP/PS1* group was significantly decreased compared with the *APP/PS1* group (*P* < 0.01, Fig. 5a), which was similar with the results of Western blot (*P* < 0.05, Fig. 5b), suggesting that EGCG treatment alleviated the overexpression of Aβ(1–40) protein in the hippocampus in *APP/PS1* mice.

**Fig. 6:**
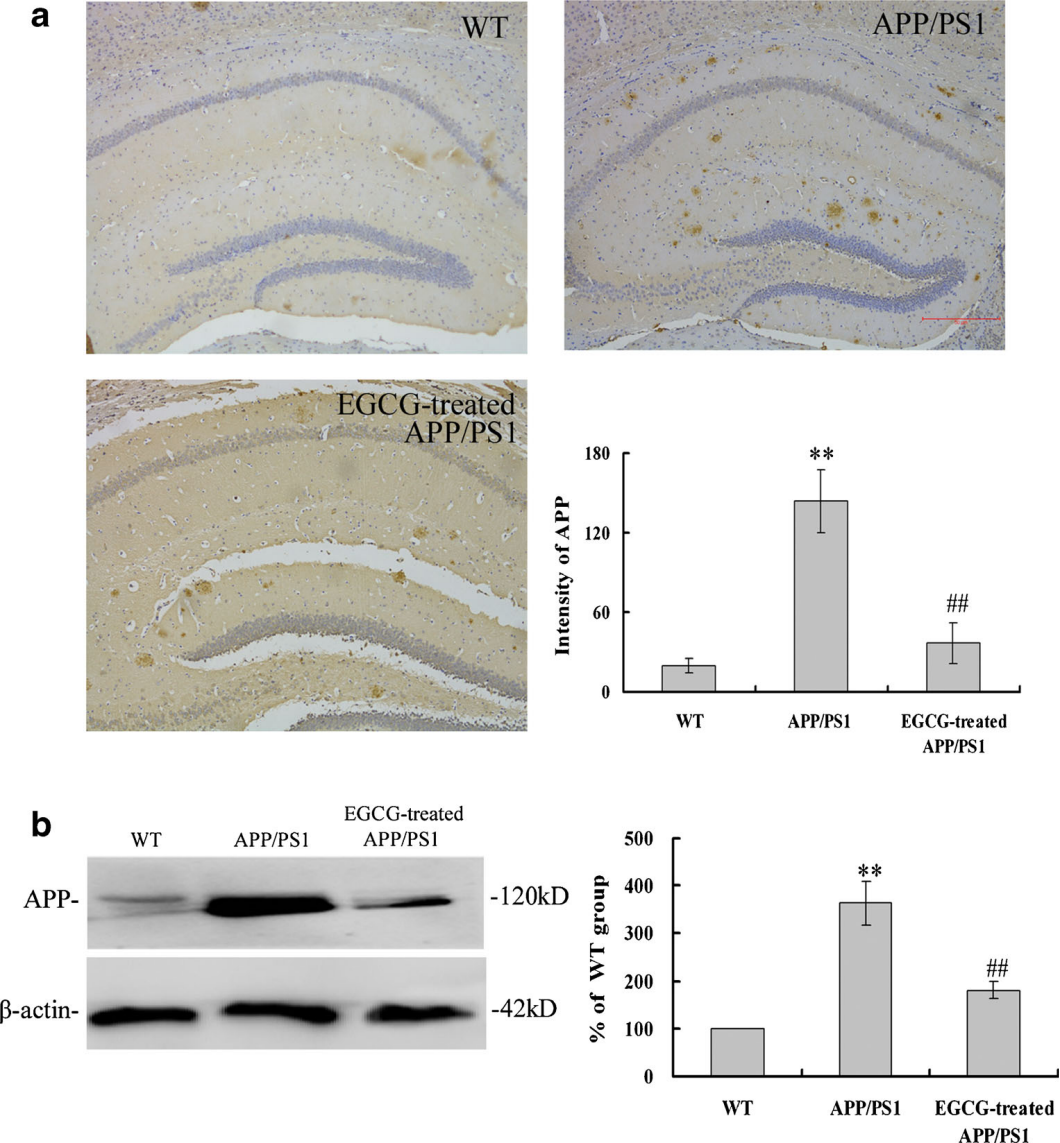
EGCG treatment decreased the APP expression levels in the hippocampus of *APP/PS1* mice by immunohistochemical staining (**a**) and Western blot (**b**) (*n* = 5). **P < 0.01, compared with WT group; ^##^ *P* < 0.01, compared with *APP/PS1* group

Furthermore, as APP is the precursor protein contributed to Aβ plaque formation associated with AD, we measured APP expression with immunohistochemistry methods (Fig. 6b) and Western blot (Fig. 6b) in *APP/PS1* mice and WT mice. As expected, *APP/PS1* mice in the *APP/PS1* group exhibited significantly higher expression of APP in hippocampus than the APP expression in the WT group (*P* < 0.01, Fig. 6a), while EGCG treatment significantly decreased the APP protein level in the hippocampus of *APP/PS1* mice in the EGCG treated group (*P* < 0.01, Fig. 6b), which were again unanimous with the results of Western blot (*P* < 0.01, Fig. 6b).

### EGCG Treatment Attenuated Neuronal Apoptosis in the Hippocampus of *APP/PS1* Mice

**Fig. 7:**
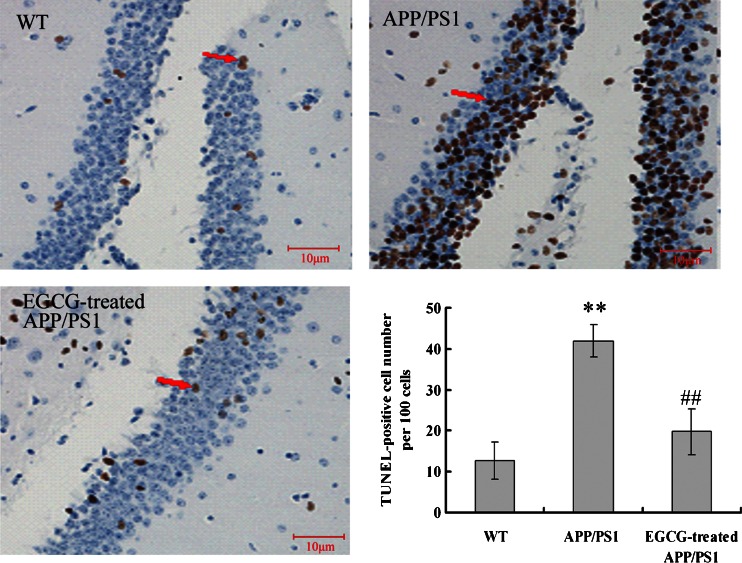
EGCG treatment ameliorated the neural apoptosis in the hippocampus of *APP/PS1* mice by TUNEL staining (*n* = 5). *Red arrow* indicated the TUNEL positive cell. *P < 0.05, **P < 0.01, compared with WT group; ^#^ *P* < 0.05, ^##^ *P* < 0.01, compared with *APP/PS1* group

Extensive Aβ(1–40) and APP deposits have been reported to induce neuron damage and neurotoxicity [3, 7, 8]. Next, we investigated whether the neuronal apoptosis was induced with overexpressed Aβ(1–40) and APP proteins in the *APP/PS1* mice. The sections of the mouse brain were stained with TUNEL method to detect apoptosis. The number of TUNEL-positive cells was significantly increased in the hippocampus (*P* < 0.01, Fig. 7) in the *APP/PS1* group compared with those in the WT group. Mice in the EGCG-treated *APP/PS1* group showed fewer TUNEL-positive cells than mice in the *APP/PS1* group (*P* < 0.01, Fig. 7).

**Fig. 8:**
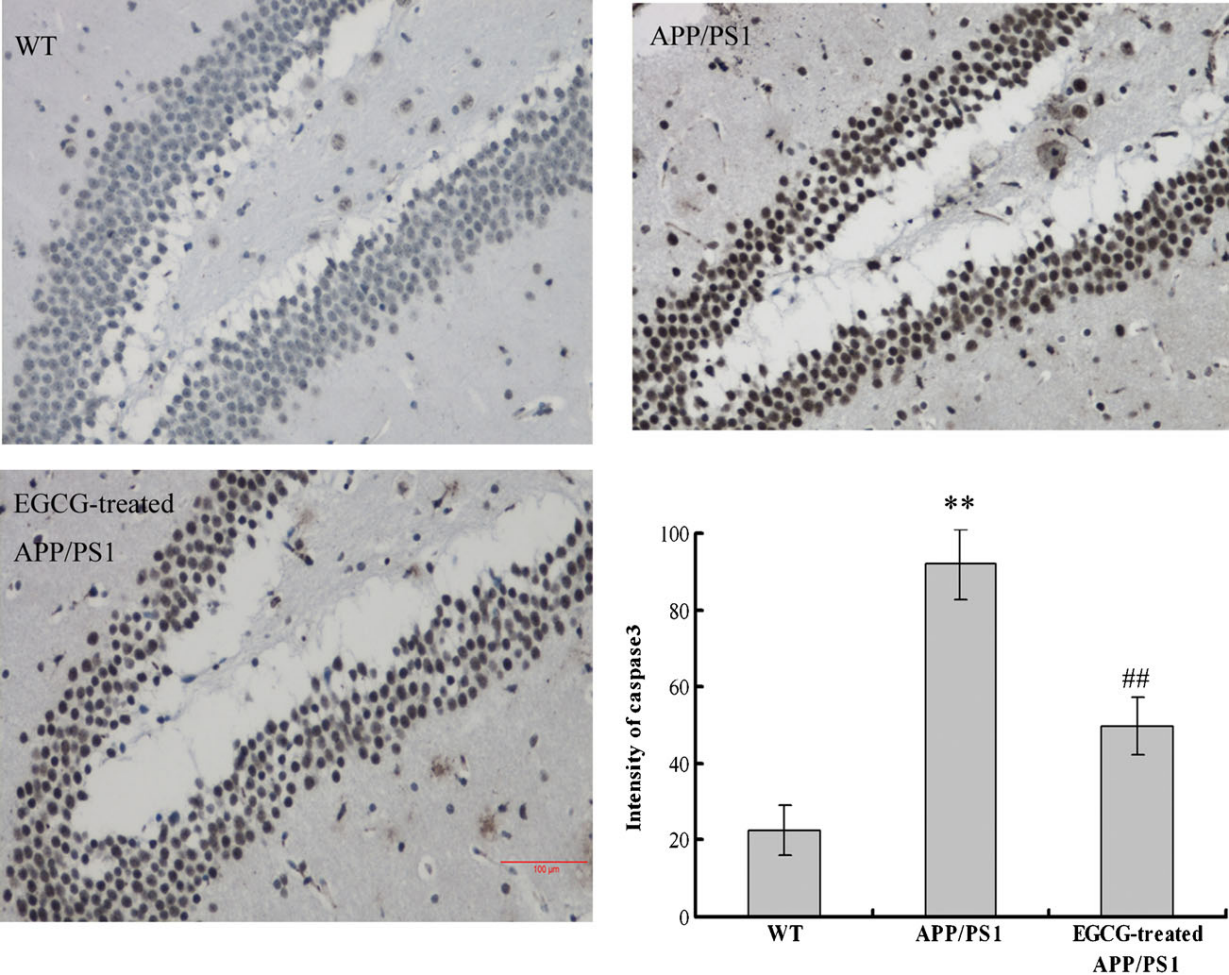
EGCG treatment decreased the expression level of caspase 3 in the hippocampus of *APP/PS1* mice by immunohistochemical staining (*n* = 3). *P < 0.05, **P < 0.01, compared with WT group; ^#^ *P* < 0.05, ^##^ *P* < 0.01, compared with *APP/PS1* group

As caspase 3 is a typical hallmark of apoptosis, we also determined the expression levels of caspase 3 by immumochemical staining for further confirmation. Concord with the TUNEL results, EGCG-treated *APP/PS1* group showed less expression level of caspase 3 than *APP/PS1* group (*P* < 0.01, Fig. 8).

**Fig. 9:**
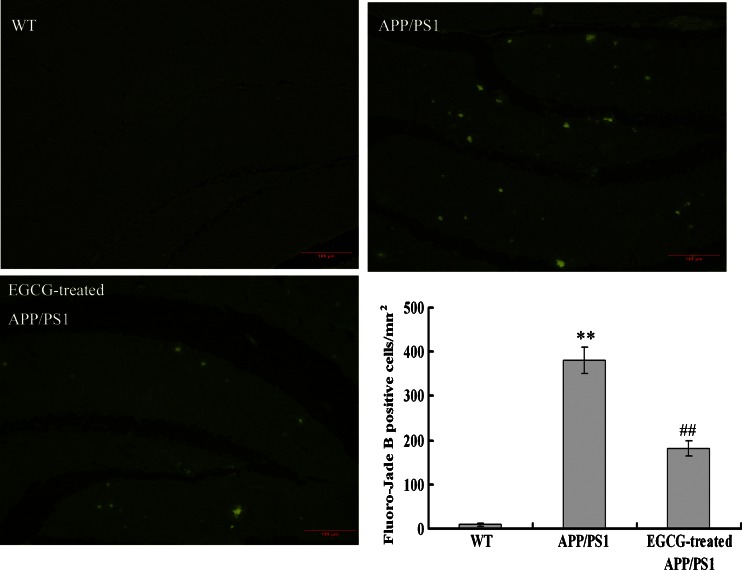
EGCG treatment improved neurodegeneration in the hippocampus of *APP/PS1* mice by Fluoro-Jade B staining (*n* = 3). *P < 0.05, **P < 0.01, compared with WT group; ^#^ *P* < 0.05, ^##^ *P* < 0.01, compared with *APP/PS1* group

Moreover, we investigated whether EGCG treatment improved neurodegeneration in the hippocampus of *APP/PS1* mice by a specific method of Fluoro-Jade B staining for degeneration for triple-check, based on the findings of TUNEL and caspase 3 immunostaining. Again, we further found that the increased Fluoro-Jade B positive cells in hippocampus of *APP/PS1* mice were significantly reduced after EGCG treatment (*P* < 0.01, Fig. 9).

Consequently, our results above indicated that EGCG treatment inhibited the neuronal apoptosis and neurodegeneration in the hippocampus of *APP/PS1* mice.

### EGCG Treatment Adjusted the TrkA/p75^NTR^ Balance by Increasing the Ratio of NGF Versus proNGF in the Hippocampus of *APP/PS1* Mice

**Fig. 10:**
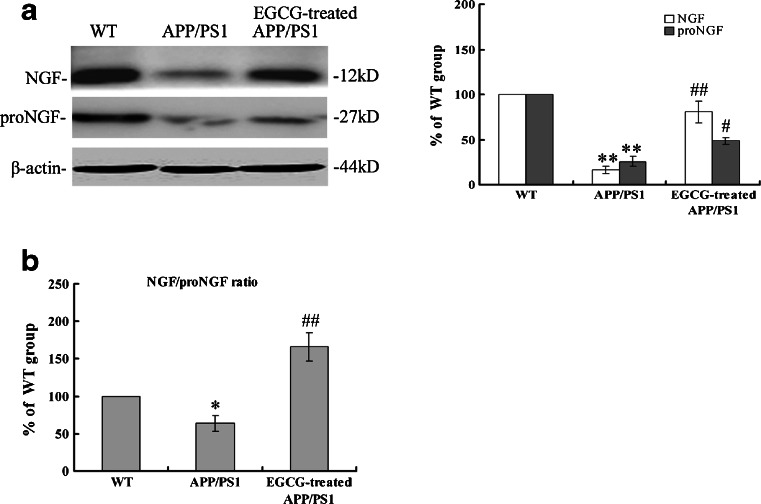
MEM treatment elevated the expression levels of NGF and its precursor neurotrophin proNGF (**a**), and increased the relative expression level of NGF versus proNGF (**b**) in the hippocampus of APP/PS1 mice (*n* = 3). *P < 0.05, **P < 0.01, compared with WT group; ^#^ *P* < 0.05, ^##^ *P* < 0.01, compared with APP/PS1 group

NGF deficiency triggers the TrkA/p75^NTR^ imbalance, which then induces apoptosis, neurodegeneration, and death [35, 36]. Therefore, we tested whether NGF and proNGF levels were altered in the hippocampus of *APP/PS1* transgenic mice with and without EGCG treatment. We found that EGCG significantly increased the levels of both NGF (*P* < 0.01, Fig. 10a) and proNGF (*P* < 0.05, Fig. 10a) in *APP/PS1* mice; furthermore, the ratio of NGF versus proNGF was also significantly increased in the EGCG-treated *APP/PS1* group (*P* < 0.01, Fig. 10b), indicating that EGCG mainly ameliorated the NGF starvation by increasing NGF level.

**Fig. 11:**
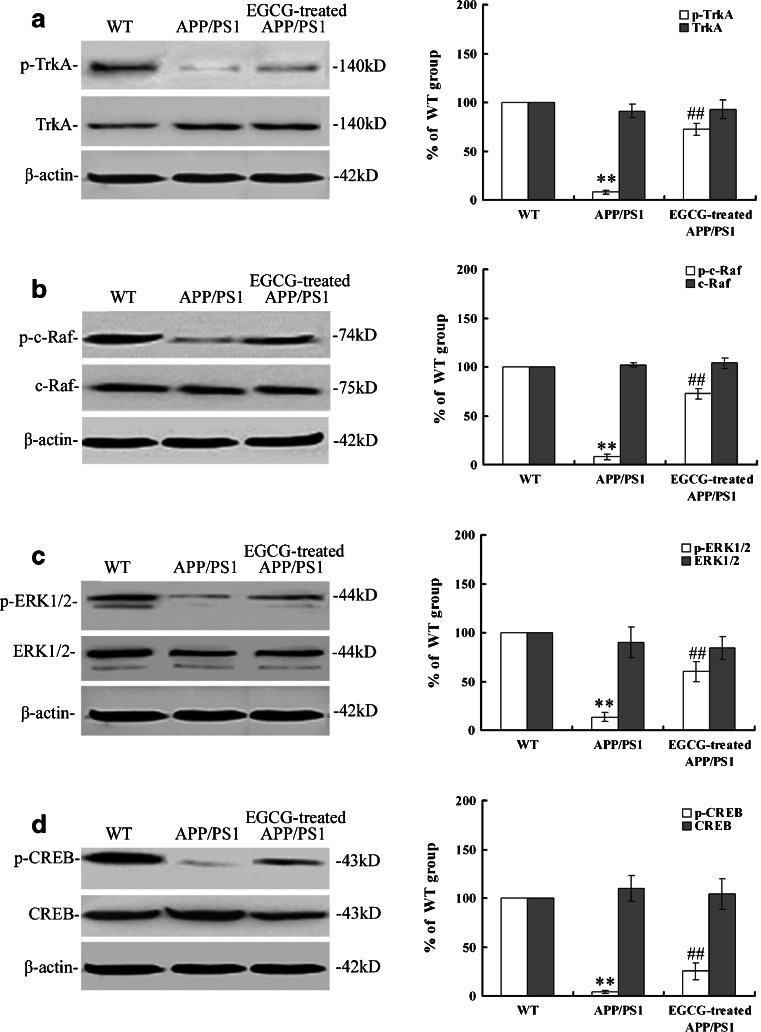
MEM treatment activated NGF-TrkA signaling by increasing the phosphorylation levels of TrkA (**a**), c-Raf (**b**), and ERK1/2 (**c**), finally upregulated the phosphorylation level of the CREB (**d**) downstream, which was closely related with memory and learning in the hippocampus of APP/PS1 mice by Western blot (*n* = 3). **P < 0.01, compared with WT group; ^##^ *P* < 0.01, compared with APP/PS1 group

Next, we investigated the NGF/TrkA-related signaling pathway and related substrates downstream in *APP/PS1* mice after EGCG treatment. After EGCG treatment, the phosphorylated protein levels of TrkA (*P* < 0.01, Fig. 11a), c-Raf (*P* < 0.01, Fig. 11b), ERK1/2 (*P* < 0.01, Fig. 11c), and CREB (*P* < 0.01, Fig. 11d) were increased, and the total protein levels were unchanged in the EGCG-treated *APP/PS1* group (*P* > 0.05, Fig. 11a–d), compared with the *APP/PS1* group, indicating that EGCG treatment activated the TrkA receptor and its downstream ERK signaling pathway by increasing NGF relative expression, and induced the activation of CREB, an important molecule for amyloidosis, learning, and memory.

**Fig. 12:**
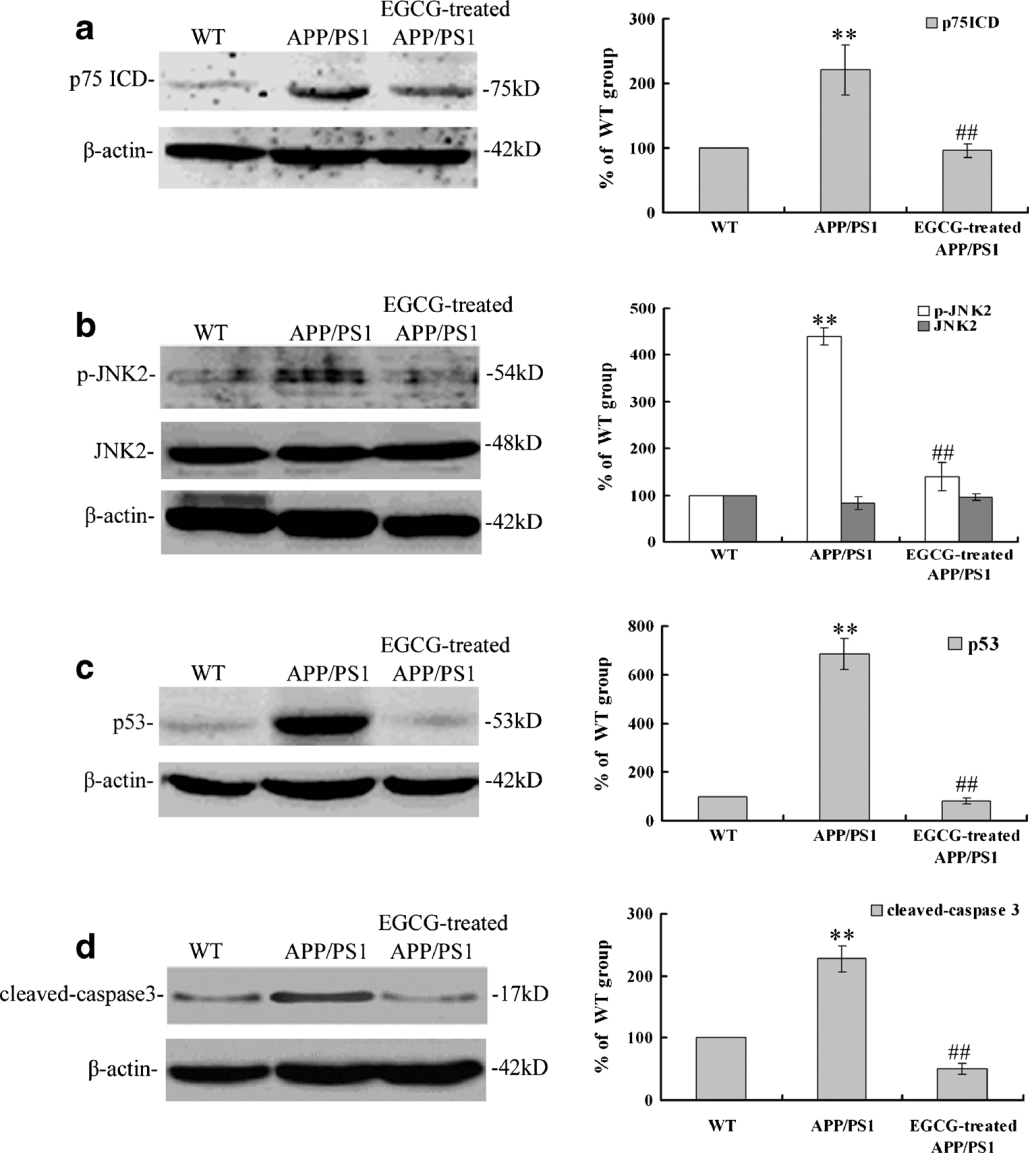
MEM treatment inhibited the cleavage ability of p75^NTR^ and its related signaling by decreasing the cleavage product p75^ICD^ (**a**), reducing the phosphorylation of JNK2 (**b**), so as to decreasing the target substrates downstream p53 (**c**) and cleaved-caspase 3 (**d**) in the hippocampus of *APPcxxPS1* mice by Western blot (*n* = 3). **P < 0.01, compared with WT group; ^##^ *P* < 0.01, compared with APP/PS1 group

Moreover, the NGF/p75^NTR^ related signaling pathway and substrates downstream in *APP/PS1* mice after EGCG treatment were also investigated in the present study. The cleavage activity of p75^NTR^ (*P* < 0.01, Fig. 12a), the phosphorylation of JNK2 (*P* < 0.01, Fig. 12b), and the expression levels of p53 (*P* < 0.01, Fig. 12c) and cleaved-caspase 3 (*P* < 0.01, Fig. 12d) were all reduced after EGCG treatment, compared with the mice in *APP/PS1* group. These results suggested that EGCG inhibited the NGF/p75^NTR^ related signaling concurring with the activiation of NGF/TrkA related signaling, and then decreased the expression levels p53 and cleaved-caspase 3, and finally inhibited the neuronal apoptosis.

## Discussions

The *APP/PS1* transgenic mouse model mimics the pathological and behavioral changes of AD based on amyloid hypothesis. *APP/PS1* transgenic mice have early onset time and intensive degree of Aβ despoition, following with neuronal loss and cognitive impairment. It is a reliable, powerful model for AD research [37]. Green tea and its polyphenolic ingredients EGCG have been suggested to possess neuroprotective effects in vitro [23–25], Lee et al. [25], Rezai-Zadeh et al. [26], and He et al. [27] reported that EGCG treatment significantly improved the cognitive deficits, APP processing, and Tau pathology in D-gal-induced AD mice, Tg*APPsw* line 2576 transgenic mice, and *PS2* transgenic mice. Jia et al. [38] recently also reported that EGCG improved memory deficits by decreasing the phosphorylation level of IRS-1; however, the neuroprotective mechanism of EGCG is still puzzled. Our results showed that EGCG treatment improved the memory and learning impairment in *APP/PS1* mice in PAT and MWM tests (Figs. 1, 2, and 3), decreased the expressional levels of APP and Aβ(1–40) (Figs. 5 and 6) and also alleviated the neuronal apoptosis and neurodegeneration (Figs. 7, 8, and 9), indicating that EGCG treatment ameliorated the cognitive deficits in *APP/PS1* transgenic mice. These results were consistent with reports in other AD mouse models including models induced by D-gal, lipopolysaccharide, or Aβ(1–42), and *PS2* transgenic mouse model [24, 39–41]. Recent studies revealed that it was the TrkA/p75^NTR^ balance contributing to the final pharmacological effects of NGF [14–16]. Therefore, we evaluated and confirmed the important role of TrkA/p75^NTR^ balance in the EGCG-induced neuroprotective effects. It is the first study to reveal that the EGCG-induced neuroprotective effect is through modulating TrkA/p75^NTR^ balance.

NGF is proposed as a functional neurotrophin stimulating cell differentiation, development, survival, and plasticity of neurons, and inhibiting apoptosis to exert nutrient and supportive effects [10, 16]. It is known that NGF elicits the survival and proliferative effects by activating the NGF/TrkA signaling, and at the same time, the NGF/p75^NTR^ signaling is suppressed by the TrkA activation to elicit the anti-apoptosis effects. On the contrary, proNGF, a precursor protein of NGF, elicits the opposite effects such as neuron loss and apoptosis by p75^NTR^ activation [14, 15]. Casponi et al. [42] found that as NGF was neutralized by the recombinant antibody in AD11 anti-NGF transgenic mice, the proNGF was predominant, which led to p75^NTR^-dependent apoptosis and cognitive deficits. The p75^NTR^-dependent apoptosis and cognitive deficits could be fully reverted by NGF administration. Therefore, the relative expression level of NGF and proNGF (that is, the ratio of NGF versus proNGF) was a key point for maintaining the TrkA/p75^NTR^ balance. The present study showed that the NGF expression level was significantly decreased (shown in Fig. 10a), and the ratio of NGF versus proNGF was also reduced (shown in Fig. 10b), suggesting that the TrkA/p75^NTR^ imbalance caused by NGF deficiency occurred in the *APP/PS1* mice. EGCG treatment significantly increased both NGF level and the ratio of NGF versus proNGF, indicating that EGCG treatment elicited the neuroprotective effects by adjusting the TrkA/p75^NTR^ imbalance.

Furthermore, we examined the TrkA signaling and the effective substrates downstream. We found that EGCG treatment ameliorated the NGF deficiency, subsequently increased the phosphorylation of TrkA receptor, activated NGF/TrkA singnaling, and increased the phosphorylation of c-Raf, ERK1/2 (shown in Fig. 11). These results agree with a recent report by Williams et al. They found that, after NGF injection, TrkA level was increased in basal forebrain, and then MAPK pathways was activated with the phosphorylation of ERK without changing the total ERK level, which indicated a possible mechanism of NGF signaling associated with aging-related memory loss in the CNS [43]. We also found that EGCG treatment increased the phosphorylation of CREB by activating NGF/TrkA signaling, in accord with the results of Autio et al. [44] that acetylcholinesterase inhibitors donepezil and galantamine increased the phosphorylation of TrkA and CREB. The activation and phosphorylation of CREB also inhibit the Aβ oligomerization and formation and decrease the Aβ neurotoxicity [45, 46]. In addition, CREB activation also reduced the messenger RNA and protein expression of γ-secretase in APP processing [47], which in turn attenuated the formation of Aβ. The attenuation of Aβ formation then ameliorated the LTP deficits and cognitive impairment and increased the synaptic plasticity [48]. Therefore, The activation and phosphorylation of CREB was associated with APP processing and Aβ desposits, which had the same tendency with our results that phosphorylation of CREB was related with the decreased levels of APP and Aβ (shown in Figs. 5 and 6). Moreover, Matrone et al. [49] found that NGF induced phosphorylation of APP by TrkA and regulated NGF/TrkA signaling, and consequentially affected the differentiation and survival of neurons. Therefore, NGF had close relationship with APP and Aβ deposits. Our results that EGCG treatment increased NGF level, decreased the expression levels of APP and Aβ(1–40), and ameliorated cognitive impairment were consistent with these observations.

Lastly, we found that EGCG decreased the cleavage activity of p75^NTR^ and inhibited the activation of NGF/p75^NTR^ signaling; then inhibited the JNK signaling and decreased the expression levels of p53 and cleaved-caspase 3 (shown in Fig. 12); and finally inhibited the neuronal apoptosis (shown in Figs. 7, 8, and 9). These results were in accord with the researches on p75^NTR^ signaling by Hashimoto et al. [50] and Costantini et al. [13], and verified the results proposed by Song et al. and Fortress et al [14, 15] that NGF/TrkA signaling and NGF/p75^NTR^ signaling were inhibited by each other.

In conclusion, EGCG treatment activated the TrkA signaling, inhibited the p75^NTR^ signaling, and adjusted the TrkA/p75^NTR^ imbalance by increasing the NGF relative expression in *APP/PS1* mice to ameliorate the behavioral deficits and amyloidosis. These results suggested that EGCG is a potential therapeutic agent to provide beneficial effects on aging and AD.
